# Improved Antioxidant Blood Parameters in Piglets Fed Diets Containing Solid-State Fermented Mixture of Olive Mill Stone Waste and *Lathyrus clymenum* Husks

**DOI:** 10.3390/antiox13060630

**Published:** 2024-05-22

**Authors:** Christos Eliopoulos, George Papadomichelakis, Arina Voitova, Nikos Chorianopoulos, Serkos A. Haroutounian, Giorgos Markou, Dimitrios Arapoglou

**Affiliations:** 1 Institute of Technology of Agricultural Products, Hellenic Agricultural Organization-DIMITRA (ELGO-DIMITRA), Sof. Venizelou 1, 14123 Athens, Greece; chris_eliopoulos@hotmail.com (C.E.); markougior@elgo.gr (G.M.); 2 Laboratory Nutritional Physiology & Feeding, Department of Animal Science, Agricultural University of Athens, Iera Odos 75, 11855 Athens, Greece; gpapad@aua.gr (G.P.); arinavoitova589@gmail.com (A.V.); sehar@aua.gr (S.A.H.); 3 Laboratory of Microbiology and Biotechnology of Foods, Department of Food Science and Human Nutrition, Agricultural University of Athens, Iera Odos 75, 11855 Athens, Greece; nchorian@aua.gr

**Keywords:** oxidative stress, weaned pigs, olive mill stone waste, *Lathyrus clymenum* husks, solid state fermentation

## Abstract

Solid-state fermentation represents a sustainable approach for the conversion of agro-industrial wastes into high-added-value feed ingredients. The present study aimed to evaluate the effects of the dietary addition of a solid-state-fermented mixture of olive mill stone waste (OMSW) and *Lathyrus clymenum* husks (LP) on the antioxidant blood parameters of weaned piglets. Two hundred 35-day-old weaned piglets were allotted into two groups and fed either a control (C) diet or a diet containing 50 g of OMSW-LP per kg (OMSW-LP) for 40 days. Blood samples were collected at 35 and 75 days of age to assess the free radical scavenging activity (FRSA), reduced glutathione (GSH) levels, catalase activity (CAT), protein carbonyls (CARBs), and thiobarbituric acid reactive species (TBARS). The OMSW-LP diet reduced the TBARS (*p =* 0.049) and CARB contents (*p =* 0.012) and increased the levels of FRSA (*p =* 0.005), GSH (*p =* 0.040), and CAT activity (*p =* 0.012) in the piglets’ blood, likely due to the synergistic action of the antioxidants and bioactive compounds present in the OMSW-LP mixture. Overall, the dietary inclusion of solid-state-fermented OMSW-LP at 50 g/kg could potentially serve a bio-functional purpose since it enhanced the antioxidant blood parameters in this study, a crucial factor for the health and growth of piglets post-weaning.

## 1. Introduction

Weaning is a period associated with health issues in piglets due to multiple social, nutritional, and environmental factors as well as inadequate management strategies [1,2]. The isolation from the sows, the adaptation to new environments, and the transition from milk to a solid feed intake enhance oxidative stress (OS), with adverse impact on piglets’ health [3,4] and, consequently, on growth, thereby negatively affecting the efficacy of commercial pig farming [5].

Several studies have recommended the supplementation of diets with feedstuffs rich in antioxidants and bioactive compounds in order to mitigate the issues posed by the OS and to protect the health of weaned piglets [6,7,8,9]. In this aspect, agro-industrial wastes may present an intriguing case because, generally, they contain a plethora of bioactive compounds [10]. The by-products that are generated from different agro-industrial processes, such as olive oil production, form ideal candidate supplements for exploitation in the sector of animal feeding since they provide the animals with the necessary energy and fiber content as well as biofunctional compounds. Several studies have revealed their beneficial effects in cattle [11], small ruminants [12,13], broilers [14], and rabbits [15]. However, their exploitation as feed additives in small ruminants displayed moderate results due to the high content of residual fat. Olive oil by-products have also been used in Iberian pigs without indicating any negative effects on growth rate or slaughter weight. On the contrary, they have displayed a positive effect on the backfat fatty acid profile due to the presence of high amounts of oleic acid and the parallel reduction in saturated fatty acids [15,16,17]. In another study, the administration of a silage containing olive mill, winery, and cheese-making by-products from a Greek industry revealed a positive impact on the health and meat quality parameters of weaned pigs. Specifically, the chemical composition of the silage was characterized by the presence of some bioactive molecules that exerted beneficial effects on gut function and microbial balance as well as on the meat’s oxidative stability [18].

However, most agro-industrial by-products are characterized by high nutrient variability, and, additionally, they are rich in structural polysaccharides such as cellulose and lignin, which are of a low digestibility. These issues complicate their potential incorporation into piglet diets. Hence, their nutritional features must be improved through the development and application of suitable methods capable of exploiting the bioactive compounds they contain. One such method is solid-state fermentation (SSF), involving the use of suitable microorganisms, such as white-rot basidiomycetes, which have the ability to secrete enzymes essential for degrading the respective structural polysaccharides [19]. These enzymes are classified into two categories: hydrolytic enzymes capable of breaking down cellulose and hemicellulose, and oxidative enzymes suitable for lignin degradation [19,20]. On the other hand, SSF initiated by white-rot fungi such as *Pleurotus ostreatus* is applied in order to address mainly nutritional issues. This includes the enhancement of the protein content, the enrichment of the substrate with various bioactive compounds like β-glucans, and, finally, the modification of the fiber content profile through lignin degradation [3].

The SSF of agro-industrial by-products also holds significance in terms of the circular economy (CE). Annually, the generated amounts of agro-industrial waste and by-products in the European Union reaches 367 million tons [10,21]. These amounts are usually disposed into nearby fields, or they are incinerated, thus leading to serious environmental issues such as soil contamination, greenhouse gas emissions, etc. [10]. The seasonable nature of most agricultural products constitutes an additional aggravating factor, since large amounts of waste are discarded in a short period of time into the environment [22,23]. Hence, SSF can contribute to overcoming the nutritional and disposal issues of these agro-industrial by-products and support an economic model targeting the efficient exploitation of natural resources [20].

In a previous work, we exploited the utilization of a mixture of agro-industrial by-products, namely 80–20% *w*/*w* olive mill stone waste (OMSW) and *Lathyrus clymenum* pericarp (LP) as a substrate for SSF initiated by *P. ostreatus* [20]. Our results showed that the SSF process increased the protein content by 58%, reduced the lignin content by 14%, and enriched the substrate with a significant amount (6.14%) of bioactive compounds such as 1,3–1,6 β-glucans, which are known for their immunostimulant and antioxidative activities. In the present study, we used the fermented 80–20% *w*/*w* olive mill stone waste (OMSW) and *Lathyrus clymenum* pericarp (LP) mixture as a feed supplement in weaned piglets, and we assessed several OS indicators to investigate whether it serves a bio-functional purpose.

## 2. Materials and Methods

### 2.1. Solid-State Fermentation of Olive Mill Stone Waste (OMSW) and Lathyrus clymenum pericarps (LP)

An 80–20% *w*/*w* mixture of OMSW and LP substrate was transformed into feed supplement in accordance with a previously described procedure by Eliopoulos et al. [20]. Briefly, the OMSW and LP substrate was prepared by weighing and mixing it in a ratio of 80% OMSW and 20% LP. The prepared substrate was placed in industrial vessels and sterilized at 121 °C for 15 min. Then, the substrate was inoculated in a vertical laminar flow chamber with the addition of a *P. ostreatus* strain at a proportion of 3% *w*/*w*, and the mixture was placed into an incubator at 25 °C in the absence of light for 21 days. During the incubation, the critical parameter of the moisture content was regulated every day by hydrating the substrate to maintain an optimum level of approximately 60%.

### 2.2. Animals and Diets

A total of 200 mixed-sex [(Large White × Landrace) × Duroc] piglets, weaned at 35 days of age, were selected and kept at a commercial farm (Keladitis farm, Pissonas, Evoia Island, Greece). The piglets were allotted into 2 experimental treatments, namely, a control (C) group and an OMSW-LP group, with 10 replicates (pens) per treatment (10 piglets/replicate) balanced for body weight (average BW of 8.12 ± 0.51 kg; mean ± S.D.). The ratio of females to males was 1:1 in each pen. The piglets were allowed a 4-day gradual transition from the commercial pre-starter diet to the experimental diets (from 35 to 38 days of age).

In the control (C) treatment, the piglets were fed a corn–soybean-meal-based diet with no additions. In the OMSW-LP treatment, the solid-state-fermented mixture of olive mill stone waste and *L. clymenum* pericarps (OMSW-LP) was added to the diet at a level of 5%. The fermented OMSW-LP mixture was added to the diet at the expense of wheat bran. The diets were formulated according to the NRC nutrient recommendations [4] and had similar crude protein and metabolizable energy contents (Table S1). The piglets had free access to feed and water throughout the experiment.

### 2.3. Experimental Procedures

Each pen was weighed at 35 (onset of the trial) and 75 days (end of the trial) of age to calculate the average daily weight gain of each treatment. The feed intake was not recorded due to practical difficulties (the experiment was carried out in a commercial farm); hence, the feed conversion ratio was not calculated. A total of 100 blood samples per treatment were collected from 5 pigs per pen (50 samples at 35 days of age, and 50 samples at 75 days of age) to assess their antioxidant blood parameters. Blood samples (4 mL) were collected from the anterior vena cava and then placed in ethylenediamine tetra-acetic acid (EDTA) tubes. The collected samples were immediately centrifuged at 1370× *g* for 10 min at 4 °C for plasma isolation. The precipitate, which represented the packed erythrocytes, was immediately lysed by the addition of distilled water (1:1 *v*/*v*). Then, the samples were inverted vigorously, centrifuged at 4020× *g* for 15 min at 4 °C, and the red blood cell lysate (RBCL) was collected.

### 2.4. Chemical Analyses

Prior to analysis, the samples of the experimental diets were ground through a 1 mm screen in a laboratory mill (CT 293 Cyclotec, Foss, Hilleroed, Denmark). The moisture, crude protein, ash, crude fiber, ether extract, and cellulose and lignin contents were determined according to the AOAC [24]. Total and reducing soluble sugars were measured in their respective aqueous extracts. In specific, the assessment of total soluble sugars was performed according to the method described by Dubois et al. [25], whilst reducing soluble sugars were evaluated according to the method reported by Miller [26]. Finally, 1,3–1,4 and 1,3-1,6 β-glucans were assessed using a MEGAZYME enzymatic assay kit (β-Glucan Assay Kit Mixed Linkage with product code: K-BGLU, and Yeast and Mushroom with product code: K-YBGL).

### 2.5. Determination of Oxidative Stress Biomarkers

Plasma samples were used for the determination of the free radical scavenging activity (FRSA), thiobarbituric acid reactive substances (TBARS), and protein carbonyls (CARBs). More specifically, the FRSA was determined according to the method reported by Janaszewska and Bartosz [27], and CARBs were evaluated according to the procedure of Patsoukis et al. [28]. The presence of TBARS, which is considered as an indicative biomarker of lipid peroxidation, was assessed herein using a modification of the method described by Keles et al. [29]. The collected RBCL samples were used for the assessment of reduced glutathione (GSH) and catalase (CAT) activity according to the methods of Reddy et al. [30] and Aebi [31], respectively.

### 2.6. Statistical Analysis

Data were analyzed using the SPSS statistical software (version 17.0). Prior to analysis, the data were tested for normality using the Kolmogorov–Smirnov test. Dependent variables that were not normally distributed were transformed according to a two-step approach, which applied an inverse–normal transformation to form a variable consisting of normally distributed z-scores [32]. Thereafter, a linear mixed model with diet as the fixed effect, age as the repeated factor, and their interactions, as well as pen as a random effect, was used to analyze the data. All data were expressed as least squares means ± root mean square error (LMSE) unless otherwise stated. To further explore any significant diet by age interactions, data were analyzed (separately for 35 and 75 days of age) by a *t*-test with diet as a fixed effect. Pen was the experimental unit, and statistical significance was set at *p* < 0.05 for all analyses.

## 3. Results

The ADF and ADL contents in the OMSW-LP diet were increased by 40% and 110%, respectively, compared to the control diet. This was expected, since the tested OMSW-LP mixture had a high lignocellulosic content (543 g/kg DM). The 1,3-1,6 β-glucans in the OMSW-LP diet were increased by 25.28% compared to the control diet (216.5 vs. 172.8 g/kg DM).

The average body weight (ABW) and the average daily weight gain (ADWG) of the piglets were not affected by the dietary treatments. At 75 days of age, the average body weight in the C and OMSW-LP piglets was 42.45 kg and 41.35 kg, respectively. The ADWG in the C and OMSW-LP piglets throughout the experiment was 0.857 and 0.831 kg/day, respectively.

Table 1 summarizes the effects of diet and age and their interactions on the parameters tested in the plasma and red blood cell lysates of the piglets. Significant differences were observed in the studied parameters between the dietary treatments. The OMSW-LP diet significantly increased the FRSA (*p =* 0.005) in the plasma and the CAT activity (*p =* 0.012) and GSH concentration (*p =* 0.040) in the red blood cells. The CARB content and TBARS concentration were significantly lower (*p =* 0.012 and *p =* 0.049, respectively) in the plasma of pigs fed the OMSW-LP diet.

**Figure 1:**
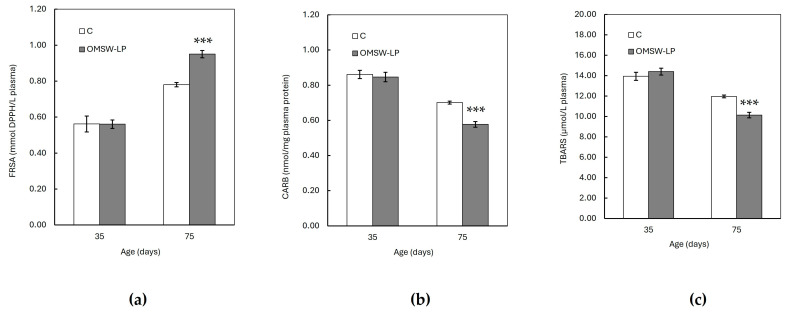
Effect of diet on plasma (**a**) total antioxidant capacity (TAC), (**b**) carbonyl (CARB) concentration, and (**c**) thiobarbituricacidreactive species (TBARS) concentration of piglets at 35 and 75 days of age (mean ± standard error of means). C, control (C) diet with no additions; OMSW-LP, diet with 50 g of solid-state-fermented mixture of 80% olive mill stone waste (OMSW) and 20% *Lathyrus clymenum* pericarps (LP) added per kg. Vertical lines represent the standard error of means. ***, statistically significant difference between diets (*p* < 0.001; two-tailed significance of the *t*-test).

**Figure 2:**
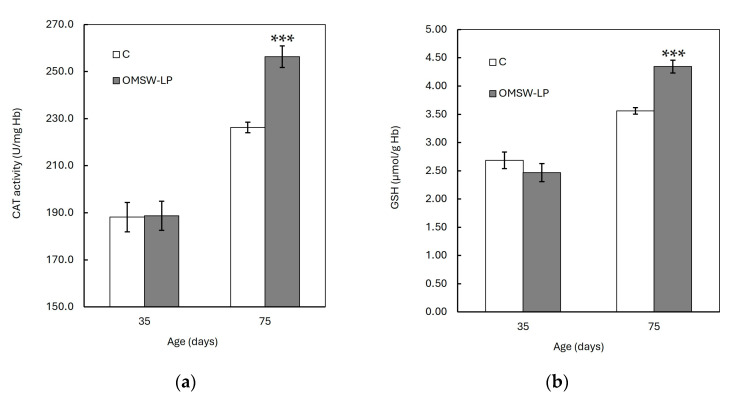
Effect of diet on red blood cell (**a**) catalase (CAT) activity and (**b**) glutathione (GSH) concentration of piglets at 35 and 75 days of age (mean ± standard error of means). C, control (C) diet with no additions; OMSW-LP, diet with 50 g of solid-state-fermented mixture of 80% olive mill stone waste (OMSW) and 20% *Lathyrus clymenum* pericarps (LP) added per kg. ***, statistically significant difference between diets (*p* < 0.001; two-tailed significance of the *t*-test).

The FRSA, CAT activity, and GSH concentration were increased (*p* < 0.001), whereas the CARB and TBARS concentrations were found to be decreased (*p* < 0.001) with age in both dietary treatments. However, these age effects were different between the C and OMSW-LP-fed piglets, as indicated by the significant diet by age interactions (*p* = 0.004, *p* = 0.004 and *p* < 0.001, *p* < 0.001 and *p* < 0.001 for FRSA, CAT activity, GSH concentration, CARB content and TBARS concentration, respectively; Table S1). To understand these interactions, the means of the oxidative biomarkers in the two dietary treatments were plotted against age (Figure 1 and Figure 2). It was observed that from 35 to 75 days of age, the FRSA (Figure 1a), CAT activity (Figure 2a), and GSH concentration (Figure 2b) increased in both groups, but the increase in the OMSW-LP group was much higher compared to that in the C-fed piglets (69% vs. 39%, 36% vs. 20%, and 76% vs. 33% for FRSA, CAT activity, and GSH concentration, respectively). It was also noted that the CARB (Figure 1b) and TBARS concentrations (Figure 1c) decreased in both groups with age, but both biomarkers exhibited a much more pronounced reduction in the OMSW-LP piglets compared to the C piglets (32% vs. 19% and 30% vs. 14% for CARB content and TBRAS concentrations, respectively).

## 4. Discussion

Weaning involves various social and environmental changes that negatively affect the antioxidant blood parameters in piglets. The removal from the sow and the transition from liquid to solid diets result in increased OS levels that cannot be effectively addressed by the underdeveloped antioxidant defense of piglets [9]. The antioxidant defense is characterized by the limited ability to scavenge free radicals and is associated with the occurrence of gastrointestinal disorders and high morbidity [33,34,35], resulting in poor growth performance [36,37] as well as high mortality in the early post-weaning period [35]. Several studies have recommended the supplementation of diets with feedstuffs rich in antioxidants and bioactive compounds to mitigate the issues posed by the OS in weaned piglets [6,7,9].

In a previous study, we showed that solid-state fermentation initiated by *P. ostreatus* can enhance the nutritive value of a mixture consisting of 80% olive mill stone waste and 20% *Lathyrus clymenum* husks (OMSW-LP) by increasing the concentration of bio-functional compounds such as 1,3-1,6 β-glucans [38]. In this work, we aimed to investigate the potential effects of the addition of dietary OMSW-LP at 50 g/kg on the antioxidant blood parameters of weaned piglets. The inclusion of OMSW-LP increased the dietary 1,3-1,6 β-glucan content by 25% in comparison with the control diet. *P. ostreatus*, which was used to carry out the solid-state fermentation process, is characterized by the high presence of β-glucans [38]. β-Glucans form a group of important bioactive compounds with potential antitumor and immune-stimulating properties, promoting human and animal health as well as welfare [39]. Chiozi et al. [40] reported that the dietary addition of β-glucans derived from mushrooms improved the immune system and egg quality in hens. In fish, enhanced immune system function, lysozyme activity, bursting respiration, and phagocytic activity were observed following the administration of dietary β-glucans [41]. β-glucans from mushrooms have also been found to possess significant antioxidant properties [42].

Oxidative status hinges on the balance between pro-oxidant and antioxidant molecules. Pro-oxidants, like free radicals generated by the mitochondrial respiratory chain, require depletion due to their potential to cause damage in living organisms, including DNA, lipid, and protein oxidation, leading to cellular and tissue injuries [3]. Their presence may also contribute to toxicity, triggering diarrhea episodes [35]. Conditions such as immune activation, physical exercise, or stress can amplify free radical generation, which exceeds the organism’s antioxidant capacity, resulting in OS [34]. In the present study, dietary supplementation with OMSW-LP strongly affected the biomarkers that are related to antioxidant capacity (FRSA, GSH, and CAT) and oxidative damage (CARBs from protein oxidation and TBARS from lipid peroxidation).

The FRSA, an indicative marker of the antioxidant content, was found to be increased for both the C and OMSW-LP piglets with age; however, the increase was significantly higher in the OMSW-LP piglets, indicating stronger free radical scavenging [43] owing to the enhanced antioxidant profile [34] of the OMSW-LP-containing diets. OMSW contains significant amounts of polyphenols, such as hydroxytyrosol, which are known to scavenge free radicals directly and elevate FRSA in pigs [44]. The solid-state fermentation that was applied herein also enriched the OMSW-LP mixture and, consequently, enriched the OMSW-LP diet with 1,3-1,6 β-glucans, which also exhibit important antioxidant properties [42]. Hence, the observed increase in the FRSA of the OMSW-LP piglets in the present study was likely a synergistic effect of polyphenols and 1,3-1,6 β-glucans on free radical scavenging. CAT is one of the most important antioxidant enzymes, acting as a decomposition agent of H_2_O_2_ to H_2_O and O_2_ in erythrocytes [45,46], thereby promoting cell protection from reactive oxygen species [47]. Its activity was significantly increased in the OMSW-LP-fed piglets because of the enhanced antioxidant profile of the OMSW-LP diet. The limited CAT activity during post-natal life makes piglets more susceptible to diseases like rotaviral enteritis, which is responsible for morbidity and mortality [34,48]. Thus, the observed increase in CAT activity herein was strong evidence of the benefits of feeding piglets with OMSW-LP. GSH is the predominant non-protein thiol in cells, which serves as a crucial antioxidant, preventing damage from OS [45]. Conditions like aging, tissue damage, and OS trigger the production of highly toxic free radicals, necessitating the initiation of antioxidant mechanisms to prevent potential toxicity to cellular components and diarrhea incidences in piglets [49,50]. Superoxide dismutase (SOD) effectively converts O_2_ to H_2_O_2_, which is then eliminated by CAT and glutathione peroxidase (GSH-Px) [51]. According to Zhu et al. [9], an increased GSH-Px content plays an important role in converting oxidized glutathione (GSSG) to its reduced form (GSH) during cellular metabolism [34]. Therefore, the elevated GSH content herein was likely the result of modifications in key enzymes responsible for GSH’s production (including enhanced GSH synthase and gamma-glutamylcysteine synthase (γ-GCL), along with increased GSH-Px activity) [45,52], indicating more resilient antioxidant defense mechanisms in the pigs fed the OMSW-LP diets.

Besides enhancing antioxidant mechanisms, the OMSW-LP diet mitigated damages caused by OS, specifically lipid peroxidation and protein oxidation. During the period of weaning, piglets are more vulnerable to OS due to the significant changes in their environment and their diet [53]. According to the existing literature, the weaning stage has been attested as a stressful period that is responsible for causing OS [9]. OS levels are increased in the early life of piglets due to their high metabolic activities essential for growth, which result in the production of reactive oxygen species (ROS). Specifically, the damages that are related with OS are considered as a consequence of the excess presence of ROS, whereas a low or transient content of the latter has the potential to trigger cellular proliferation mechanisms or to promote survival via signaling pathways [54]

Lipid peroxidation generates degradation products like malondialdehyde (MDA), which is capable of negatively affecting pig performance and health [55]. Plasma TBARS, a key biomarker of lipid peroxidation, was observed to decrease in the OMSW-LP-fed piglets, indicating more effective antioxidant mechanisms compared to the C-fed piglets. The enhanced antioxidative defense in the OMSW-LP-fed piglets was also reflected in the decreased protein oxidation, as evidenced by the significant reduction in the CARB content. Protein oxidation can lead to the polymerization and aggregation of proteins, resulting in the loss of their function with negative effects on cellular activities; hence, its prevention is of crucial importance [45,56]. The reduced CARB content may be linked to the concomitant increase in GSH activity [34,44,57] in the OMSW-LP-fed piglets.

Besides the effects on the dietary bio-functional compound concentration, the addition of the OMSW-LP increased the dietary ADF (cellulose and lignin) content by 37% compared to the control diet. Cellulose and lignin can generally be considered antinutritional factors because they are negatively correlated with nutrient digestibility and growth performance. However, no such effect was observed herein; both the C- and OMSW-LP-fed piglets had comparable daily weight gains. Previous works reported that feeding diets with a high cellulose content post-weaning had beneficial effects on piglet health and growth performance, as they reduced digestive disorders and mortality rate [58]. Also, improved immunological parameters [59], intestinal barrier function [60], and limited intestinal pathogen proliferation have been observed in weaned piglets fed high-cellulose diets [60].

## 5. Conclusions

This is the first study to investigate the use of a solid-state-fermented mixture of agro-industrial wastes as a feed ingredient rich in bioactive compounds and its effects on the antioxidant blood parameters of weaned piglets. The administration of diets with 50 g of a solid-state-fermented OMSW-LP mixture/kg for 40 days post-weaning significantly improved the antioxidant blood parameters, a crucial factor for the health and growth of piglets post-weaning. This was evidenced by the enhanced antioxidant defense mechanisms (increased FRSA in plasma and GSH content and CAT activity in red blood cells) and the reduction in the levels of lipid (TBARS) and protein (CARB) oxidation products in plasma. Our findings can be attributed to the synergistic effects of antioxidants and bioactive compounds (such as polyphenols and 1,3-1,6 β-glucans) present in the OMSW-LP mixture on free radical scavenging. Overall, the transformation of agro-industrial wastes into added-value products with bio-functional compounds and their use in feeding can be a valuable tool to enhance the antioxidant blood parameters of weaned piglets. It can also be an effective strategy towards environmental sustainability and conservation in the context of a circular economy.

## Figures and Tables

**Table 1:** Effects of diet and age on plasma free radical scavenging activity (FRSA), carbonyl (CARB) and thiobarbituric-acid-reactive species (TBARS) concentrations, red blood cell lysate catalase (CAT) activity, and glutathione (GSH) concentration.

	Diet (D) ^a^		RMSE ^b^	Age (A)		RMSE ^b^	*p*-Values		
	C	OMSW-LP		35d	75d		D	A	DxA
Plasma									
FRSA (mmol DPPH/L)	0.67	0.76	0.028	0.56	0.87	0.028	0.005	0.001	0.004
CARB (nmol/g)	0.78	0.71	0.026	0.86	0.64	0.011	0.012	0.001	0.001
TBARS (μmol/L)	12.96	12.20	0.283	14.17	11.05	0.248	0.049	0.001	0.001
Red blood cell lysate									
CAT (U/mg Hb ^c^)	207.21	222.80	5.593	188.47	241.54	4.582	0.012	0.001	0.004
GSH (μmol/g Hb ^c^)	3.12	3.40	0.146	2.58	3.95	0.104	0.040	0.001	0.001

^a^ C, control diet; OMSW-LP, diet with 50 g of solid-state-fermented mixture of 80% olive mill stone waste (OMSW) and 20% *Lathyrus clymenum* pericarps (LP) added per kg. ^b^ Root mean square error. ^c^ Hemoglobin.

## Data Availability

Data is contained within the article.

## Supplementary Materials

The following supporting information can be downloaded at: https://www.mdpi.com/article/10.3390/antiox13060630/s1.
