# Supplementary material for: Improved Antioxidant Blood Parameters in Piglets Fed Diets Containing Solid-State Fermented Mixture of Olive Mill Stone Waste and Lathyrus clymenum Husks
Source: Antioxidants (Basel). 2024 May 22;13(6):630. doi: 10.3390/antiox13060630 (PMC11201101; doi:10.3390/antiox13060630)
Supplement: Supplementary file 1 [file antioxidants-13-00630-s001.zip › antioxidants-2961449-supplementary.pdf]

Supplementary Materials

Table S1. Ingredient (g/kg as fed) and chemical composition (g/kg dry matter) of the experimental diets.

	Diet[a]	
	**Control (C)**	**OMSW-LP**
Ingredient		
Maize	585.0	610.8
Soybean meal (480 g CP/kg)	225.0	245.0
Wheat bran	100.0	-
OMSW/LP	-	50.0
Soybean oil	45.8	50.0
Calcium carbonate	12.0	9.0
Monocalcium phosphate	9.0	12.0
Sodium chloride	6.2	6.2
L-Lysine HCl 80%	6.0	6.0
DL-Methionine 99%	3.0	3.0
L-Threonine 99%	3.0	3.0
Mineral + vitamin premix[b]	5.0	5.0
Analyzed chemical composition		
Dry matter (g/kg)	880.0	885.5
Ash	65.5	64.0
Crude protein	197.8	197.4
Ether extract	85.5	88.7
Crude fiber	40.0	58.2
ADF	49.3	67.8
ADL	9.1	19.4
1,3-1,6 β-glucans	172.8	216.5
Calculated chemical composition		
Digestible energy (MJ/kg DM)	16.3	16.2
Metabolizable energy (MJ/kg DM)	15.6	15.6
Cellulose (ADF – ADL)	40.2	48.4
Calcium	8.6	8.5
Total phosphorus	6.8	6.7
Lysine	14.8	14.8
Methionine+cystine	9.8	9.6
Threonine	10.5	10.4
SID[d] Lysine	13.5	13.6
SID[d] Methionine+cystine	9.0	8.9
SID[d] Threonine	9.3	9.2

[a] C, control; OMSW-LP, with 50 g solid state fermented mixture of 80% olive mill stone waste (OMSW) and 20% *Lathyrus clymenum* pericarps (LP) added per kg. [b] Mineral and vitamin premix provided per kg of diet: 15,000 IU vitamin A (retinyl acetate), 2000 IU vitamin D_3 (cholecalciferol), 100 mg vitamin E (DL-α-tocopheryl acetate), 3.75 mg menadione (vitamin K_3), 1.25 mg vitamin B_1, 6 mg vitamin B_2, 20 mg vitamin B_5, 1.5 mg vitamin B_6, 27.5 μg cyanocobalamin (B_{12}), 32.5 mg nicotinic acid, 1 mg folic acid, 150 μg biotin. [c] Digestible energy, macro-element and amino acid values for maize, soybean meal and wheat bran were adapted from tabulated data [4]. [d] Standardized ileal digestible aminoacid.
